# A Fiber Bragg Grating-Based Dynamic Tension Detection System for Overhead Transmission Line Galloping

**DOI:** 10.3390/s18020365

**Published:** 2018-01-26

**Authors:** Guo-ming Ma, Ya-bo Li, Nai-qiang Mao, Cheng Shi, Bo Zhang, Cheng-rong Li

**Affiliations:** 1 State Key Laboratory of Alternate Electrical Power System with Renewable Energy Sources, North China Electric Power University, Beijing 102206, China; ncepulyb@126.com (Y.L.); upc_mao2011@163.com (N.M.); ncepusc@163.com (C.S.); lcr@ncepu.edu.cn (C.L.); 2 Henan Electric Power Research Institute, State Grid, Zhengzhou 450000, China; zhangbo18@ha.sgcc.com.cn

**Keywords:** optical fiber sensors, tension, remote sensing, galloping measurement, overhead transmission lines

## Abstract

Galloping of overhead transmission lines (OHTLs) may induce conductor breakage and tower collapse, and there is no effective method for long distance distribution on-line galloping monitoring. To overcome the drawbacks of the conventional galloping monitoring systems, such as sensitivity to electromagnetic interference, the need for onsite power, and short lifetimes, a novel optical remote passive measuring system is proposed in the paper. Firstly, to solve the hysteresis and eccentric load problem in tension sensing, and to extent the dynamic response range, an ‘S’ type elastic element structure with flanges was proposed. Then, a tension experiment was carried out to demonstrate the dynamic response characteristics. Moreover, the designed tension sensor was stretched continuously for 30 min to observe its long time stability. Last but not the least, the sensor was mounted on a 70 m conductor model, and the conductor was oscillated at different frequencies to investigate the dynamic performance of the sensor. The experimental results demonstrate the sensor is suitable for the OHTL galloping detection. Compared with the conventional sensors for OHTL monitoring, the system has many advantages, such as easy installation, no flashover risk, distribution monitoring, better bandwidth, improved accuracy and higher reliability.

## 1. Introduction

Overhead transmission lines (OHTLs) are an important part of a power grid which easily suffer from the impact of complex meteorological and geographical conditions [1,2,3]. Many online monitoring systems were developed to improve the reliability of OHTLs, such as temperature monitoring [4], sag monitoring [5], and degraded compression and bolted joints monitoring [6,7]. Galloping of overhead transmission lines is a common wind-induced vibration with a low frequency and high amplitude, which occurs in both single and bundle conductors. The galloping may reduce the air gap clearances between conductors, occasionally leading to flashover, and repeated power supply interruptions [8]. Moreover, the galloping of the iced conductor and the corresponding weight increase may also stress the conductor joints/splices and, as a consequence, may lead to the conductor breakage and tower collapse [9]. CIGRE summarized 192 reports on galloping from 28 countries, and tens of millions dollars have been spent to repair the lost electrical infrastructure [3]. Thus, this is an important design and operational problem for electric utilities.

The phenomenon has been investigated for many years from the theoretical and experiment viewpoints, and many measures have been developed [10,11]. However, as galloping occurs in different situations, the anti-galloping devices that may work well on one site might actually increase the probability of occurrence of galloping at another site [3]. The key problem is that the galloping behaviors of transmission lines in different situations have not been obtained accurately. Because experiments are useful to investigate a mathematical model of conductor galloping or perform an experimental validation of devices to prevent flashovers [11,12], an effective on-line remote galloping monitoring system is extremely needed, especially for OHTLs located in mountains where galloping often happens.

Video, acceleration sensors and voltage sensors have been tried for field galloping observation. (1) Video cameras are installed in remote locations to monitor galloping. Motions of the image of the conductor across the video screen are detected and transmitted by a GPRS network [13]. However, as the span of the OHTL is usually 100 m to 500 m, the video is not able to cover a complete span, and the lifetime of the cameras is limited in the harsh environment. (2) Acceleration sensors are mounted the phase conductor. For example, ten acceleration sensors are installed separately on 400 m span. Beside the difficulty of the field installation, the galloping behaviors cannot be calculated accurately based on the acceleration data due to the fact the conductor may rotate during the galloping. In addition, the acceleration sensors are powered by batteries that limits their working time. (3) Tensions can be used to obtain the galloping behaviors of phase conductors [3], and conventional voltage sensors based on strain gauges are located between insulators and towers [14]. However, the method still has some disadvantages for long-term monitoring. Firstly, because of lack of the field power, the sensors are often solar powered, but the solar electric charger may fail after continuous ice days when the galloping is severe. Secondly, the sensors are easily disturbed by the strong electromagnetic field as they are installed beside the high voltage transmission lines. Thirdly, field experiences indicate that the lifetime of the sensors is not as good as expected.

In our research, we wanted to develop a novel optical remote passive galloping measuring system. In China, many optical fiber composite overhead ground wires (OPGWs) have been installed above phase conductors, especially on the high voltage transmission lines. In our research, an optical tension sensor is developed to replace the conventional voltage sensor. The optical tension sensor is installed on the electric tower, and the interrogator is put in the substation that tens kilometers away. The optical signal is transmitted in a fiber in the OPGW. Thus, the power supply, electromagnetic interferences, and lifespan problems mentioned above all can be solved.

To date, sensors based on fiber Bragg gratings have been successfully developed for many areas, such as monitoring infrastructure, composite materials, pipelines, biochemical and electrochemical experiments and many other aspects [15,16,17,18,19,20,21]. Thus, we investigated the galloping sensor based on the FBG principle. For the on-line monitoring of overhead transmission lines, Bjerkan investigated vibrations of overhead lines by gluing a FBG strain sensor on the phase conductors [22], and Huang et al. clamped a FBG strain sensor on the phase conductor to measure its tension [23]. These studies are instructive, but both the two sensors are too fragile to be used in the field and hard to install. Hao et al. developed a distributed on-line temperature and strain fiber sensing system based on the combined Brillouin optical time domain reflectometry (BOTDR) and fiber Bragg grating (FBG) technology [24], and distribution measurement was achieved with the system, but the BOTDR is only used to measure the temperature of the OPGW, as a steel tube is used to protect the fiber and the purpose is to reduce the tension of the inner fiber of OPGW. The load of the OPGW could not be transferred to the inner fiber and the BOTDR technique cannot be used to measure the tension along the OPGW [24]. Besides that, the galloping behaviors of OPGW and phase conductor are different, including frequencies, vibration modes and amplitudes [25].

In the paper, a FBG tension sensor is designed to measure force variations caused by galloping. Firstly, the measurement principle and sensor design is introduced in detail. Secondly, under the wind force, the phase conductor with ice gallops fast. Observations in the field indicate that the high frequency cut-off frequency of galloping is about 3 Hz [3]. Thus, a tension experiment is carried out using a high force electromechanical tester to demonstrate the dynamic response characteristics from 0.2 Hz to 3 Hz (sensor bandwidth). Then, the designed tension sensor is mounted on a fatigue testing machine and stretched continuously for 30 min (1800 times) for observing its long time stability. Finally, to verify the feasibility of the proposed on-line monitoring system, a series of experiments have been carried out at the State Grid Key Laboratory of Power Overhead Transmission Line Galloping (Zhengzhou, China). Different vibration patterns were generated by the galloping testing machine during the experiments. The experimental results demonstrate the sensor is suitable for OHTL galloping detection. Compared with the conventional electric sensors, the advantages of the proposed sensor are anti-electromagnetic interference, no power supply requirement, distribution measurement and long life in a humid environment. Different from sensors developed in [22,23], the proposed sensor is mounted on the zero potential conductor other than a high voltage conductor. Thus, to the best of authors’ knowledge, the proposed sensors can be installed without the line power being off, at least in China [26], making the installation process much easier. On the other hand, the accuracy and reliability of the developed sensor is higher because of the special structure design. Besides that, if we want to link the sensors in [22,23] with an OPGW for remote and distribution sensing, an optical fiber must be located near the insulator surface which reduces the flashover voltage of the insulator and may induce an interruption of power supply. As the sensor proposed is located on the zero potential tower, there is no insulation risk. Compared with the sensor described in [24], the accuracy of the proposed sensor is much higher, because an elastic element structure with flanges was designed to solve the hysteresis and eccentric load problem in traditional tension sensors of the column type, and to extend the dynamic response range.

## 2. Measurement Principle of Optical Tension Sensor

**Figure 1:**
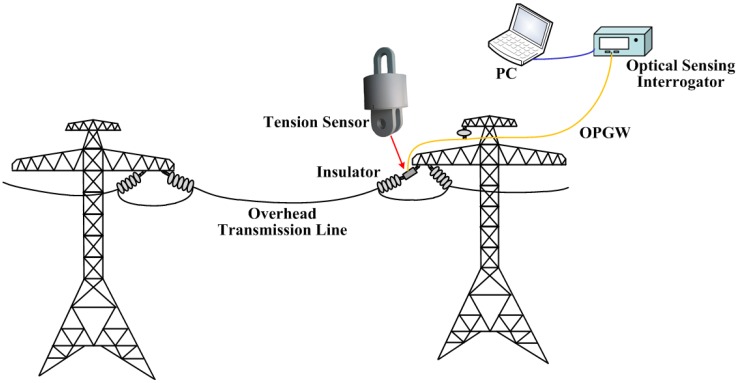
The mounting position of the optical tension sensor.

A dynamic tension detection system was developed to detect the overhead transmission line galloping. The system composes of FBG tension sensors, an optical fiber composite overhead ground wire (OPGW) and a wavelength interrogator. A schematic diagram of the system is shown in Figure 1.

The FBG tension sensor was designed to measure the dynamic tension of a phase conductor, and it is installed between an electric tower and an insulator string which is used to hang the phase conductor. Then, an optical signal of the sensor is transmitted back to the wavelength interrogator through a fiber in the OPGW. Finally, wavelengths shifts of FBGs caused by the dynamic tension are demodulated by the wavelength interrogator, and then the tension between the tower and the insulator strings is calculated based on the output wavelength. With the Wave-Division Multiplexing (WDM), the system can simultaneously measure the galloping occurred at different spans. In the future, based on the wavelength scanning time division multiplexing technique of ultra-weak fiber Bragg gratings (FBG) [27], the galloping behaviors of a tens kilometers overhead transmission line can be detected and presented.

**Figure 2:**
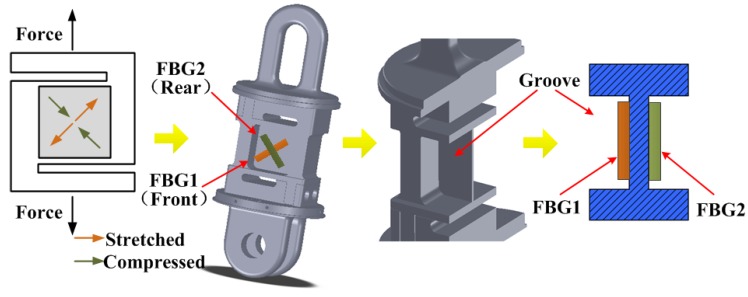
Elastic element structure and FBGs locations of the tension sensor.

For linking the electrical towers and insulator strings conveniently, we amended the top and bottom shapes of the elastic elements specifically referring to the traditional fitting of PH-10 and U-10 as Figure 2 shows. Because there are two connection points on the bottom of the elastic element. Thus the load applied on the elastic element is eccentric. Thus, the structure of the elastic element must be investigated to reduce the influence of the eccentric load. The explanations was added in the revised paper. An elastic element structure with flanges was proposed to solve the hysteresis and eccentric load problem in traditional tension sensor of the column type, and to extent the dynamic response range. As illustrated in the Figure 2, the shape of elastic element of the optical tension sensor is the shearing structure of S type, while the cross-section of the elastic element was designed as the I-shaped to obtain a nearly uniform strain distribution on the parts where the FBGs measured. The material of the elastic element is 35 CrMnSiA. Besides that, the grooves of the elastic element are effective to balance the distribution of strain on it and improve the accuracy. Moreover, the top and bottom shapes of the elastic elements were specially chosen to become suitable for the link fittings of the electrical tower and insulator string.

Two fiber Bragg grating strain gauges, which are packaged in the metallic materials, are mounted on the different grooves with the direction deviation of 90° from each other to eliminate the temperature effect and improve the accuracy. The metal substrate of the fiber grating strain gauge is welded to the elastic element to solve the problem of poor strain transfer effect caused by the traditional strain adhesive. Moreover, it also improves the long-term stability.

When a tension variation caused by galloping applied on the elastic element of the optical tension sensor, the optical strain gauges mounted on the surface of the grooves are subjected to different strains which induces FBG wavelength shifts are equal in value but opposite in direction. As one FBG is stretched and the other FBG is compressed, the shifts in FBG reflective wavelengths ($\Delta \lambda_{FBG1},\;\Delta \lambda_{FBG2}$) affected by the applied strain variations ($\Delta \varepsilon_{1},\;\Delta \varepsilon_{2}$) and the temperature variation (ΔT) can be described as:

(1) $$\begin{array}{l} \Delta \lambda_{FBG1}\;=\;K_{S}\Delta \varepsilon_{1}\;+\;K_{T}\Delta T\\ \Delta \lambda_{FBG2}\;=\;K_{S}\Delta \varepsilon_{2}\;+\;K_{T}\Delta T \end{array}$$

where $K_{S}$ is the strain coefficient (pm/με), $K_{T}$ is the temperature coefficient (pm/${}^{{}^{\circ}}C$).

Thus the wavelength separation shift of the two FBGs is:

(2) $$\Delta \lambda \;=\;\Delta \lambda_{FBG1}\;-\;\Delta \lambda_{FBG2}\;=\;K_{S}\left(\Delta \varepsilon_{1}\;-\;\Delta \varepsilon_{2}\right)\;\propto \;F$$

As indicated in (2), the wavelength separation shift of two FBGs is independent from the temperature but proportional to the tension caused by galloping. The cross-sensitivity of FBGs to temperature variation is overcome by using the built-in differential structure.

**Figure 3:**
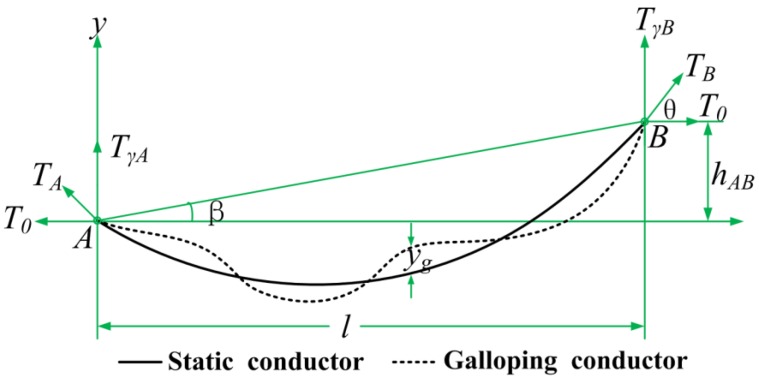
The model of conductor galloping with different height suspension points.

With the tension measured by the optical sensor, we can obtain frequencies, modes and amplitudes of galloping conductors. Many scholars have established the relationship between wire dancing and dynamic tension change in the form of theoretical deductions. The line length method is the most commonly accepted method used to derive the dynamic tension change of the transmission line caused by the galloping dancing [28,29]. As illustrated in Figure 3, the shape of the static conductor is shown as the solid line and *l* is the length of the conductor in span (m), the *h_AB_* is the high difference of the two conductor hanging point (m) and *β* is the horizontal angle of the two conductor hanging point (°). Then, *T_A_*, *T_B_* denote the axial tension at the suspension point of the conductor respectively (N), which is along the direction of the insulator string. *T_γ__A_*, *T_γ__B_* are the vertical tension components at the suspension point of the two ends (N) and *T*_0_ is the horizontal tension component (N).

The references [29,30] indicates that one loop galloping (n = 1) and two loops galloping (n = 2) account for nearly 86.6% of the observed field galloping. Besides that, the amplitude of the higher loops galloping is much smaller. Thus, only the amplitude of one loop galloping (n = 1) and two loops galloping (n = 2) is analyzed. According to the parabolic model of the overhead transmission line, the length change of the galloping conductor is deduced as follow [28]:

(3) $$\Delta l=\{\begin{array}{ll} \frac{n^{2}\pi^{2}{a_{0}}^{2}}{4l}\sin^{2}(\omega t)-\frac{2a_{0}Wl}{n\pi T_{0}\cos\beta }\sin(\omega t) & n=1\\ \frac{n^{2}\pi^{2}{a_{0}}^{2}}{4l}\sin^{2}(\omega t) & n=2 \end{array}$$

Where *a*_0_ is the amplitude of galloping conductor (m), *n* is the number of standing wave loops in the span, *l* is the length of the conductor in span (meter), *ω* is the frequency of galloping conductor (rad/s), *t* is time (s), *W* is the load of conductor in unit length (N/m).

Due to the length of the conductor affected by the transmission line galloping, the insulator string at the tower is subjected to a dynamic tension. The relationship between the length variation of conductor and the dynamic tension can be calculated from the Hooker's Law of the elastic deformation, so the amount of change in the horizontal tension of the conductor can be expressed as:

(4) $$\begin{array}{l} \Delta T_{0}=EA\frac{\Delta l}{l}=k_{c}\Delta l\\ =\begin{array}{ll} \frac{k_{c}n^{2}\pi^{2}{a_{0}}^{2}}{4l}\sin^{2}(\omega t)-\frac{2k_{c}a_{0}Wl}{n\pi T_{0}\cos\beta }\sin(\omega t) & n=1\\ \frac{k_{c}n^{2}\pi^{2}{a_{0}}^{2}}{4l}\sin^{2}(\omega t) & n=2 \end{array} \end{array}$$

where *k_c_* = *EA*/*l*, *E* is the Young modulus of the conductor (N/mm^2^), *A* is the cross-sectional area of the conductor (mm^2^).

The amplitude is determined from the tension and Equation (4). Firstly, the parameters about the specific transmission line is obtained from the design company or the power utility. Then, the tension and the frequency of galloping conductor is calculated based on the sensor measurement result.

Thirdly, the number of standing wave loops can be estimated with the amplitude spectrum of tension measured. For one loop galloping (n = 1), because sin^2^ (*ωt*) = (1−cos2*ωt*)/2, there are two dominant oscillation frequencies in the amplitude spectrum of tension measured, and one oscillation frequency is twice the frequency of the other (as indicated in Equation (4)). For two loops galloping (n = 2), there is only one oscillation frequency. The estimation procedure of the number of standing wave loops are also explained in the section about the experiment result. Finally, with the parameters obtained in the above three procedures, the amplitude of the galloping can be calculated based on Equation (4).

## 3. Dynamic Experiment of Tension Sensor

### 3.1. Tension Sensor Dynamic measurement

**Figure 4:**
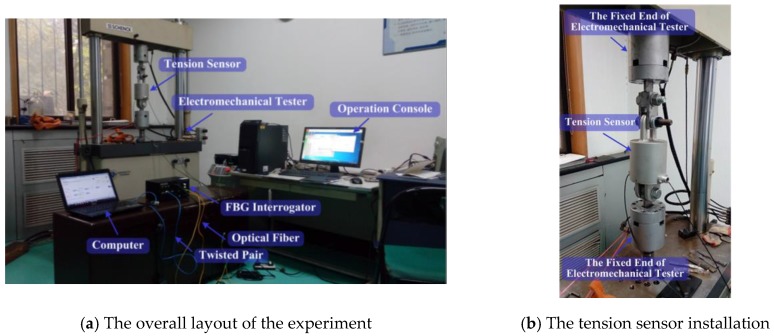
The setup of the dynamic stretched experiment.

Under wind forces, phase conductors with ice may gallop. As mentioned in the Introduction, the working range requirement of the system is 0.2–3 Hz. Obviously, a static experiment is not enough to investigate the performance of the developed tension sensor in the field galloping monitoring. Therefore, the developed tension sensor was stretched at different frequencies using a high force electromechanical tester to examine the dynamic output characters. The experimental setup is shown in Figure 4.

**Figure 5:**
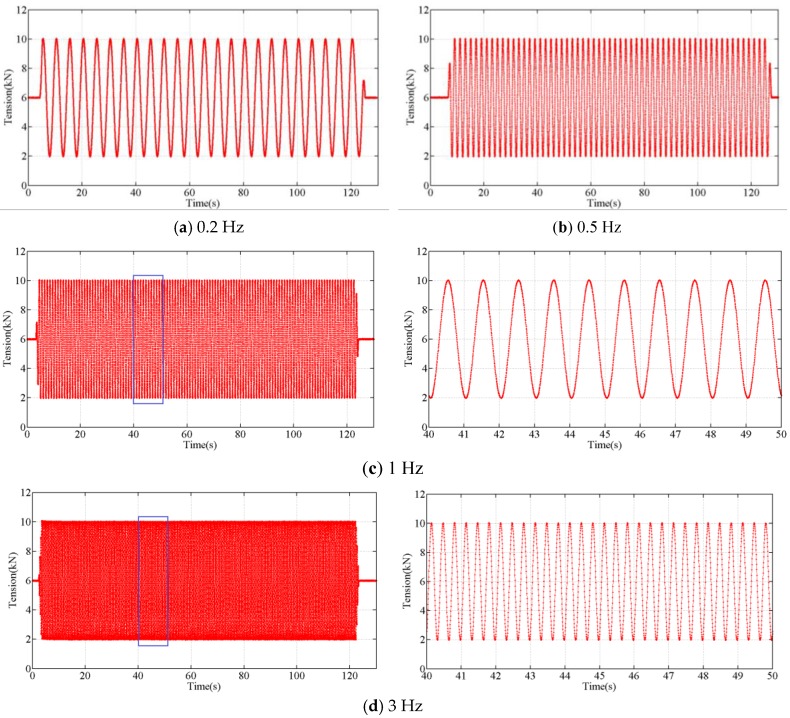
The force measured during tension experiment at different frequencies.

**Figure 6:**
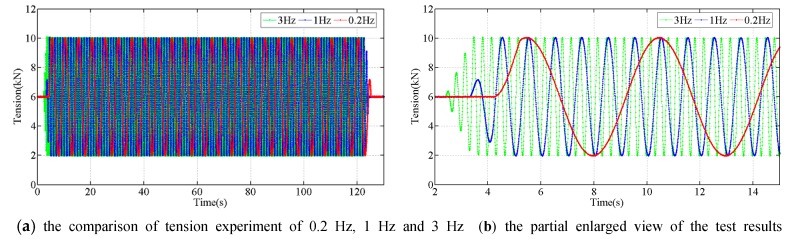
The results comparison of the dynamic tension experiments.

The proposed sensor was stretched in the form of sinusoidal waves at the frequencies from 0.2 Hz to 3 Hz, using the force amplitudes from 2 kN to 10 kN. The tension experiment at different single frequency lasted for 2 min. The tensions measured during the experiments at the different frequencies are shown in Figure 5 and Figure 6. The results of the experiments indicate that the output of the developed tension sensor is stable in the frequency range 0.2 Hz to 3 Hz.

As we already know, the low frequency accuracy of the tension sensor is easily achieved and has been demonstrated by our former experiment [30,31], the developed tension sensor has a good bandwidth and it can be used to measure the dynamic tension variations caused by a galloping conductor.

### 3.2. Tension Sensor Fatigue Experiment

**Figure 7:**
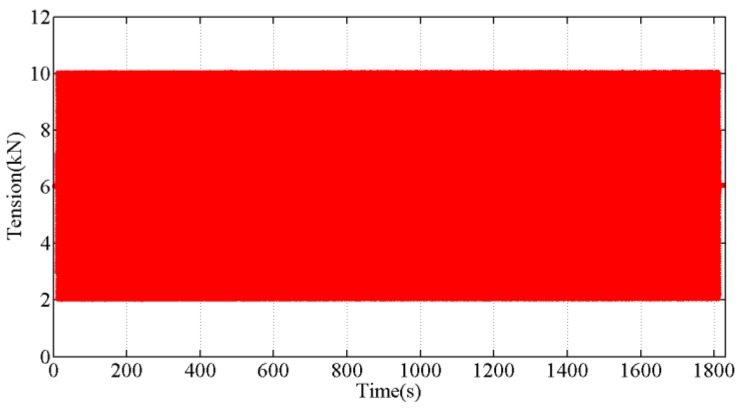
The measured tension during the sensor fatigue stretching experiment.

Long-term performance of the tension sensor is also important since the sensor must work in field for years. In material science, creep is the tendency of a metal to deform permanently under the influence of mechanical stresses, it indicates the long-term performance of the sensor. In the part 6.5 of standard OIML R60: 2013 “Metrological Regulation for Load Cells” [32] for electric load cells, the test time for a creep observation is 30 min. Thus, the developed tension sensor was mounted on a fatigue testing machine and stretched continuously for 30 min (1 Hz, 1800 times) for the purpose of observing whether the sensor output would change. The output tension curve of the sensor during the fatigue experiment is shown in Figure 7, and there is no shift in the measuring range.

**Figure 8:**
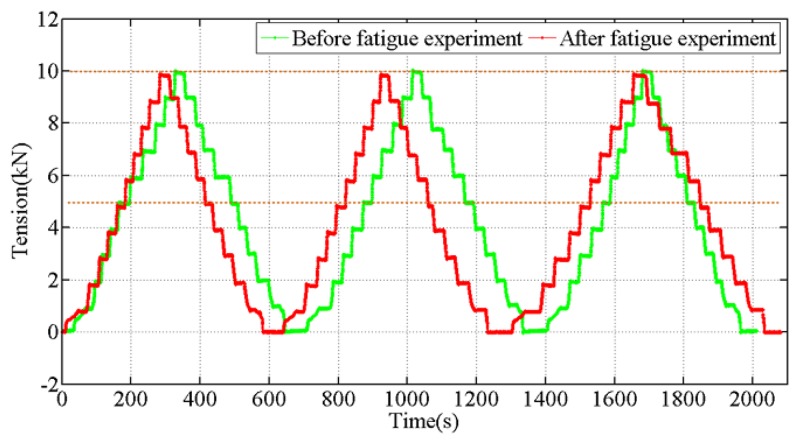
The performance comparison of tension sensor before and after fatigue experiment.

Two tension step experiments were carried out before and after the fatigue test separately. For the test, the interval times between different steps were not the same. As shown in Figure 8, the results of the experiments indicate that accuracy of the designed tension sensor is not affected by fatigue tension cycles.

## 4. Galloping Experiment on a Transmission Line Model

### 4.1. Galloping Experiment Setup

**Figure 9:**
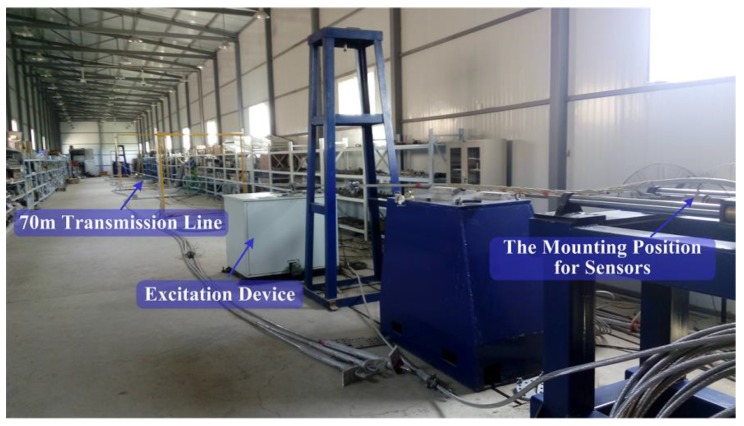
The setup of galloping experiment on transmission line model.

A conductor galloping experiment was carried out on a transmission line model, provided by the State Grid Key Laboratory of Power Overhead Transmission Line Galloping (Zhengzhou, China). As shown in the Figure 9, the total length of the conductor model installed on the experiment platform is 70 m. The excitation device generates an external force that causes the conductor to galloping, and the galloping frequency can be adjusted at multiple frequencies, such as 0.5 Hz, 1.03 Hz.

**Figure 10:**
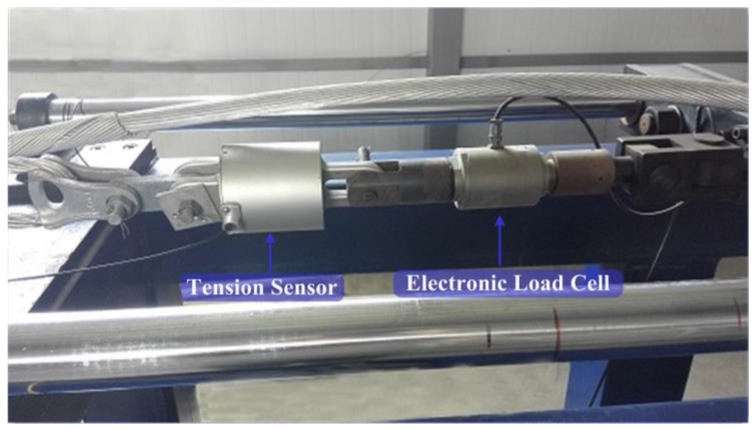
The connection diagram of tension sensors.

During the experiment, the horizontal dynamic tensions produced by the transmission line model galloping were recorded by an electronic tension sensor and the optical tension sensor. We installed the optical tension sensor at one end of the transmission line model, which was connected in series with the electronic tension sensor, which are illustrated in Figure 10. The sampling frequency in the experiment was set to 20 Hz.

### 4.2. Experiment Results and Discussion

**Figure 11:**
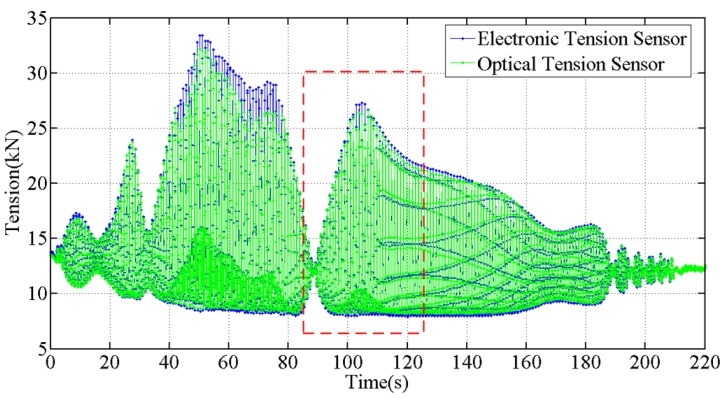
The tension measured during the galloping (frequency is 1.02 Hz), the curve in the red dashed box is shown in Figure 12.

**Figure 12:**
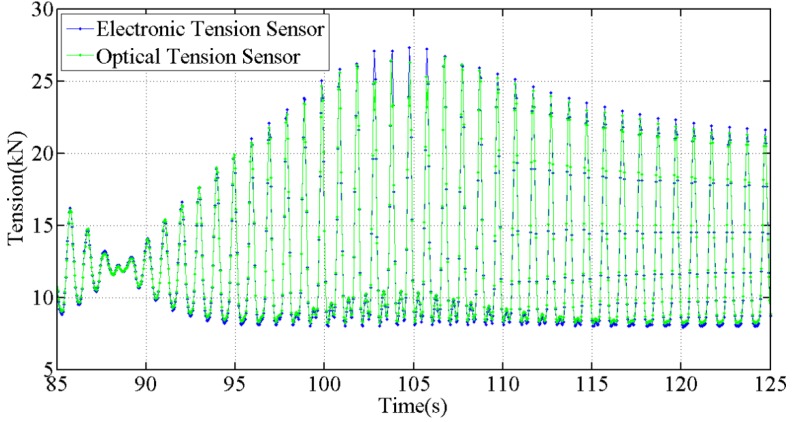
Tension measured by the optical sensor in the period of 85 s to 125 s, drive frequency is 1.02 Hz.

At the beginning of the galloping experiment, we recorded the initial wavelengths of the optical strain gauges mounted on the tension sensor, which were 1554.983 pm and 1549.889 pm. Then, the experiment platform was powered to make the transmission line model galloping. Experience shows that the inherent resonant frequency of the OHTL model is near 1 Hz, below which the conductor is prone to large-scale galloping. Thus, in the first test, the drive frequency of the experiment platform was set to 1.02 Hz. The whole process of the first galloping test included two procedures of start and stop, the tension is plotted in Figure 11 and Figure 12.

As shown in Figure 12, the output of the optical tension sensor is basically consistent with that of the electronic tension sensor, which proves that the optical tension sensor has good dynamic measurement characteristics.

**Figure 13:**
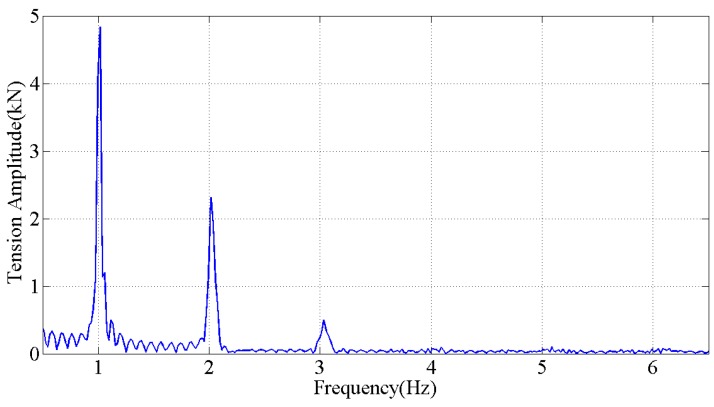
The amplitude spectrum of the tension measured by the optical sensor during the galloping (85 s to 125 s), drive frequency is 1.02 Hz, x-axis limits is 0.5 Hz to 6.5 Hz.

The fast Fourier transform (FFT) was applied to obtain the amplitude spectrum of tension measured. Figure 13 indicates that the dominant oscillation frequency is near 1 Hz, which is consistent with the drive frequency set in the experiment. The frequency is close to the natural oscillation frequency of the experimental transmission line model.

**Figure 14:**
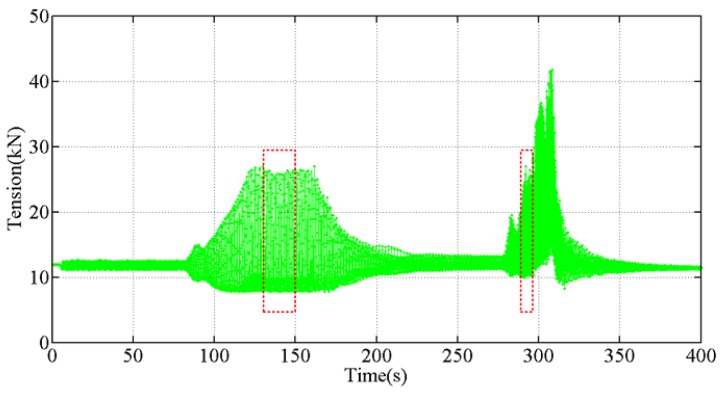
The dynamic tension results in the second galloping experiment, in which different drive frequencies were used.

The amplitudes and frequencies of a transmission overhead lines change in an actual galloping accident induced by wind conditions. In the second experiment, the drive frequency was set to 0.8–2.4 Hz to simulate galloping in the field. The force measured by the optical tension sensor is plotted in Figure 14. The parameters in Equation (4) except the amplitude of the galloping conductor (*a*_0_) and the number of standing wave loops in the span (*n*) are known. The number of standing wave loops can be estimated with the amplitude spectrum of tension measured.

**Figure 15:**
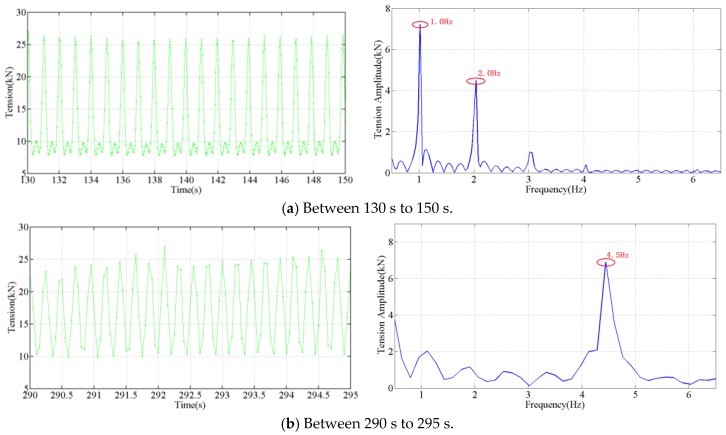
Measured tension and its amplitude spectrum (x-axis limits is 0.5 Hz to 6.5 Hz) during the galloping frequency variation experiment.

Zoomed tensions and their amplitude spectrums are illustrated in the Figure 15. Between 130 s to 150 s, the force measured and its amplitude spectrum are shown in Figure 15a. There are two dominant oscillation frequencies, 1 Hz and 2 Hz, in the measured amplitude spectrum of the tension, and one oscillation frequency (2 Hz) is twice the frequency of the other (1 Hz). According to the analysis before, it is a one loop galloping, *n* = 1.

Between 290 s to 295 s, the force measured and its amplitude spectrum are shown in Figure 15b. There is only one oscillation frequency span, According to the analysis before, it is a two loops galloping, *n* = 2.

The standing wave loops estimation procedures are illustrated by the video provided in the Supplementary Material of this manuscript, 15 s (*n* = 1) and 50 s (*n* = 2).

The developed optical tension sensor can accurately distinguish the characteristic frequency and mode of the conductor galloping, the amplitude can be determined from the tension based on Equation (4). The performance of the developed sensor is demonstrated by the dynamic measurements.

## 5. Conclusions

A novel optical passive measuring system is proposed for monitoring the galloping of overhead transmission lines by using the OPGW transmission. The benefits of the optical detection method are anti-electromagnetic interference, no power supply requirement and long life in humid environments.

An ‘S’ type elastic element structure with flanges is proposed to solve the hysteresis and eccentric load problems, and to extent the dynamic response range. The tension experiments carried out using a high force electromechanical tester demonstrate the dynamic response characteristics. Fatigue experiments verified the long term stability of the sensor.

To examine the feasibility of the proposed on-line monitoring system operated on a phase conductor, different patterns galloping were generated in a 70 m conductor model, and the experimental results demonstrated that the sensor is suitable for OHTL galloping detection.

Compared with the optical sensors for OHTL monitoring, the system has many advantages, such as easy installation, no flashover risk, distribution monitoring, better bandwidth, improved accuracy and higher reliability.

## Supplementary Materials

The supplementary materials are available online at http://www.mdpi.com/1424-8220/18/2/365/s1.
